# How does the human visual system compare the speeds of spatially separated objects?

**DOI:** 10.1371/journal.pone.0231959

**Published:** 2020-04-30

**Authors:** M. V. Danilova, C. Takahashi, J. D. Mollon

**Affiliations:** 1 Department of Psychology, University of Cambridge, Cambridge, England, United Kingdom; 2 I. P. Pavlov Institute of Physiology, St. Petersburg, Russian Federation, Giessen, Germany; Justus Liebig Universitat Giessen, GERMANY

## Abstract

We measured psychophysical thresholds for discriminating the speeds of two arrays of moving dots. The arrays could be juxtaposed or could be spatially separated by up to 10 degrees of visual angle, eccentricity being held constant. We found that the precision of the judgments varied little with separation. Moreover, the function relating threshold to separation was similar whether the arrays moved in the same, in opposite or in orthogonal directions. And there was no significant difference in threshold whether the two stimuli were initially presented to the same cerebral hemisphere or to opposite ones. How are human observers able to compare stimuli that fall at well separated positions in the visual field? We consider two classes of explanation: (i) Observers’ judgments might be based directly on the signals of dedicated ‘comparator neurons’, i.e. neurons drawing inputs of opposite sign from local regions of the visual field. (ii) Signals about local features might be transmitted to the site of comparison by a shared ‘cerebral bus’, where the same physical substrate carries different information from moment to moment. The minimal effects of proximity and direction (which might be expected to influence local detectors of relative motion), and the combinatorial explosion in the number of comparator neurons that would be required by (i), lead us to favor models of type (ii).

## Introduction

In the study of sensory processes, psychophysics still has an honorable role [1]. We need psychophysics to define the perceptual abilities that are to be explained; to place constraints on theoretical models; and to suggest the stimulus parameters that are appropriate if imaging or electrophysiology is to isolate a particular neural channel. In the present paper we use psychophysics to explore an aspect of visual perception that is theoretically interesting but seldom discussed: If two stimuli are presented at different, widely separated, positions in the visual field and if the exposure duration is too short for eye movements, how precisely can the stimuli be compared? This humble empirical issue raises fundamental questions about the format and the protocol of transmission within and between the cerebral hemispheres–questions that would be difficult to address with current imaging methods.

### The mechanism underlying the comparison of separated stimuli

Some sensory discriminations are known to deteriorate quickly as the spatial separation of the stimuli increases. This is the case for comparisons of the luminance of two patches [2], or of the relative depth of two targets [3], or of the temporal phase of two bars that are modulated in contrast [4]. In such examples, we might suppose that the observer relies on signals from local, hard-wired, neural comparators such as have been revealed by electrophysiological recording from the mammalian visual system. A paradigmatic neural comparator would be a retinal ganglion cell that draws opposed inputs–excitatory and inhibitory–from adjacent regions of the photoreceptor array. Such a unit signals local contrast to the brain [5]; and when we are asked to compare the luminances of two abutting fields, our decision may be based on edge-contrast signals that ultimately originate in such ganglion cells [6]. Similarly, cells are found in Area V2 of the macaque that respond to the relative depth of adjacent stimuli in a local region of the field [7].

**Fig 1:**
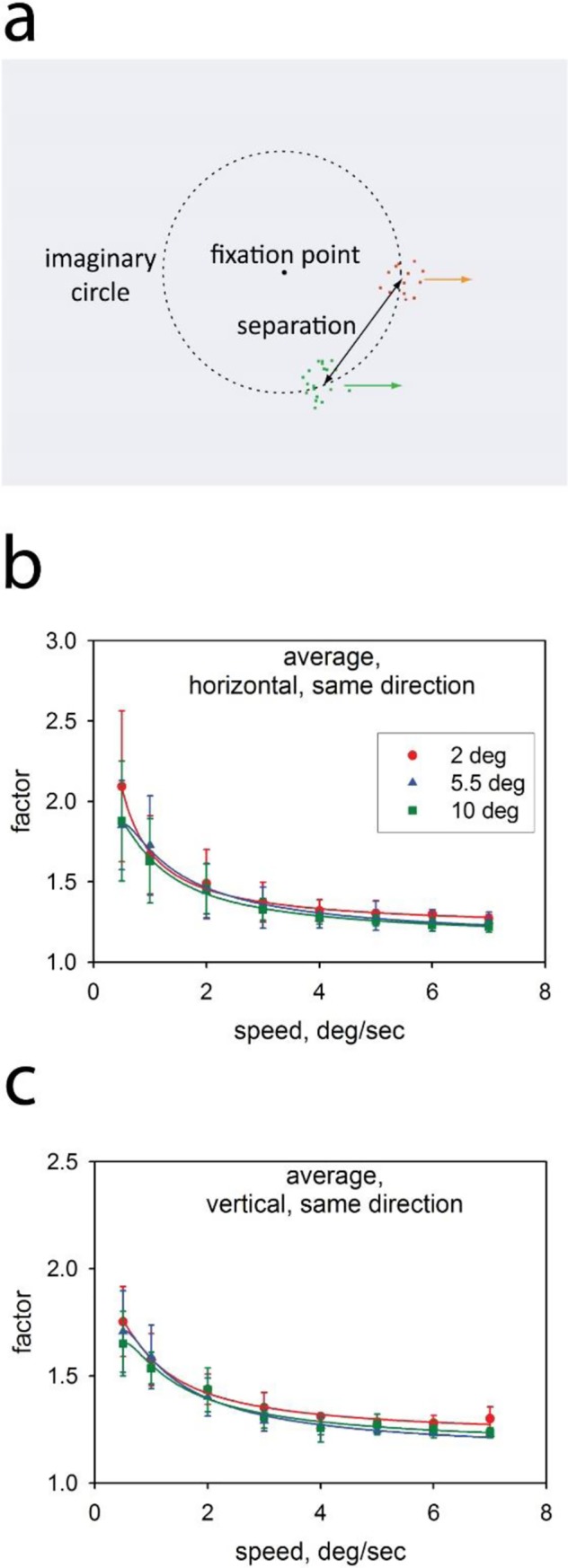
**a**. Arrangement of stimuli in the experiments. The patches of moving, pseudorandom dots always fell at random positions on an imaginary circle, 10 degrees of arc in diameter, but their spatial separation was different in different blocks of trials. The colored arrows indicate the direction of movement (in this example, horizontal). **b, c** Results from preliminary experiments where speed was varied in different blocks of trials and where different spatial separations were tested in different experimental runs. The ordinate represents the factor by which the test speed differed from the referent speed at threshold (79.4% correct) and the abscissa represents the speed of the referent stimulus. Data are the averages from five highly trained observers. Error bars represent standard errors of the mean. The fitted curves are inverse second-order polynomials and have no theoretical significance. Panel **b** shows results for horizontal motion, and panel **c** for vertical.

In the case of other visual attributes, however, such as spatial frequency, comparisons can be made with the same precision whether the discriminanda–the stimuli to be compared–are juxtaposed or whether they lie 10 degrees apart in opposite hemifields [8]. There exists no accepted neural model of how such perceptual judgments are made. It would be possible to propose a dedicated neural comparator cell for every possible pair of positions in the visual field and for each of several visual attributes. For the analysis of spatial contrast, there have been occasional suggestions of an array of “dissociated dipole” operators that perform non-local, but spatially specific, comparisons of the type envisaged [Fig 1; 9,10]. But a neural model of this kind leads to a combinatorial explosion in the number of dedicated neural comparator units required; and in the Discussion we consider an alternative possibility, that information about local attributes is carried in abstract, symbolic codes that travel over a shared bus to the site of comparison.

### The present experiments

In the series of experiments reported here, we used a 2-alternative forced choice procedure to measure sensitivity for discriminating the speeds of two arrays of moving random dots. The two arrays were briefly presented at spatially separated positions in the visual field (Fig 1A). The eccentricity of the arrays was held constant but their separation was varied.

Could performance in this task be based on dedicated detectors of contrast of speed, i.e. units that respond to a difference in the speeds present at different locations? Several psychophysical results from other paradigms do suggest the existence of sensory channels sensitive to local contrast of either the speed or the direction of motion: Examples are the abolition of the motion after-effect in the absence of a surround [11,12]; the Duncker effect (the illusory motion seen when a stationary field is enclosed by a moving framework) [13,14]; the dependence of apparent speed on the speed of adjacent elements [15]; the paradoxical reduction in direction discrimination when the size of a high-contrast target is increased [16,17]; and the visibility of static edges that are indicated only by kinetic cues [18 ch 3]. And indeed, cells sensitive to the relative speed or direction of motion in adjacent regions have repeatedly been described at different levels of primate visual systems [e.g. 19,20,21,22,23,24,25,26,27,28]. Particularly clear examples of units sensitive to *speed* contrast can be seen in Fig 2 of Allman et al [20] and Fig 13 of Allman et al [19].

The primary roles of cells sensitive to motion contrast are thought to lie in the segregation of figure from ground, in the estimation of depth and form from motion parallax, and in the analysis of optic flow fields for the maintenance of balance and the guidance of locomotion [see e.g. 19,21,29,30,31]; and not all such cells exhibit tuning for speed [21,28]. However, if the discrimination of speed did depend on signals from intrinsically local comparators of motion, we might expect performance to deteriorate with increasing spatial separation of the stimuli.

As a second test of the hypothesis that speed discrimination depends on signals from dedicated contrast-sensitive units, we also examined the effect of varying the directions of the motions in the two patches. The responses of neurons at early stages of the visual system often depend on two or more attributes, in addition to retinotopic position itself [32]. So we might expect local comparator neurons for one attribute to have a systematic preference for a second attribute. Neurons that signal speed commonly have an intrinsic preference for direction [20,33]; and in the pre-striate motion area V5 or MT (middle temporal), direction of motion is a primary basis for the columnar organization [34,35]. If, therefore, discrimination of relative speed depends on dedicated comparator neurons, the comparators are likely to draw their inputs from lower-order units that have a similar preference for direction (or for axis of direction). In that case we might expect the function relating speed thresholds to separation to have a different form according to whether or not the subsets of dots were moving in the same or in opposite or in orthogonal directions.

### Ensuring a true comparison

In studying sensory discrimination, it is important to be sure that the observer makes a true comparison rather than an absolute judgment of one of the two stimuli presented [36]. If the reference stimulus is fixed from trial to trial, then–remarkable as it seems–a trained observer may build up so accurate an internal template of the average stimulus presented that he or she performs better by attending to only one of two stimuli [37,38,39]. For the judgment is then based on only one sample of external noise rather than the two samples of external noise that would be introduced if target and reference stimuli were actively compared. Such use of an implicit standard has been shown specifically for the case of speed discrimination by Norman and colleagues [40]. We here adopted two precautions to ensure that the observer did actively compare the two stimuli. First, in our main experiments, the reference stimulus on any trial was not fixed but was jittered over a range of speeds: In this ‘roving’ condition, a single psychophysical staircase was used to adjust the ratio of test to reference speed, but the reference varied randomly over a range that was larger than the discrimination threshold. Second, in each experimental run of the main experiments, we included a control condition where the computer program randomly suppressed one or other of the discriminanda and the observer was asked to make an absolute judgment as if the second stimulus were present.

## Results

Panels b and c of Fig 1 show results from the preliminary experiments, designed to identify a range of speeds where the Weber fraction for speed discrimination was relatively constant–a range that could be used for the ‘roving’ procedure of the main experiments. In these preliminary experiments, several reference speeds were tested, without jitter, and measurements were obtained at 3 different spatial separations. In Fig 1B results are shown for motion on a horizontal axis (leftward or rightward on different trials, randomly chosen, but with both patches moving in the same direction on a given trial); and Fig 1C shows analogous results for motion on a vertical axis. The y-axis in these and subsequent plots represents the factor by which the test speed must differ from the reference speed to sustain a performance of 79.4% correct, the threshold level tracked by our staircase procedure. In agreement with previous authors [41,42,43], we find that discrimination is poorest at low speeds but there is a range of higher speeds at which the Weber fraction is approximately constant.

Already in the data of Fig 1, we see also that spatial separation has only small effects on thresholds: The functions for the three different separations are similar. For the horizontal condition, a repeated-measures ANOVA, with factors Speed and Spatial Separation, shows a highly significant effect of speed but no effect of spatial separation and no significant interaction (After Greenhouse-Geisser correction, Speed, *F*(1.191) = 29.341, *p* = 0.003, Separation, *F*(1.41) = 2.683, *p* = 0.155, Interaction (*F*(2.112) = 1.034, *p* = 0.401). In the case of vertical motion, there is again a highly significant effect of Speed (*F*(1.373) = 25.369 *p* = 0.002) and there is also a marginally significant effect of Separation, (*F*(1.174) = 9.369, *p* = 0.029)–although it is the smallest separation that yields the highest thresholds. The interaction was not significant (*F*(1.424) = 1.181, *p* = 0.356).

In the subsequent main experiments (1–6) we measured speed thresholds as a function of spatial separation, but the speed of the nominal ‘reference’ stimulus was allowed to ‘rove’ from trial to trial in the range 3.2 to 6.8 deg/s. There were 25 possible reference values, spaced at intervals of 0.15 deg/s. The ‘variable’ stimulus always had the higher speed but differed from the reference by a factor that was adjusted according to a single staircase. Using the same procedures throughout, we ran 6 independent experiments, which differed in how different directions of motion were combined.

**Fig 2:**
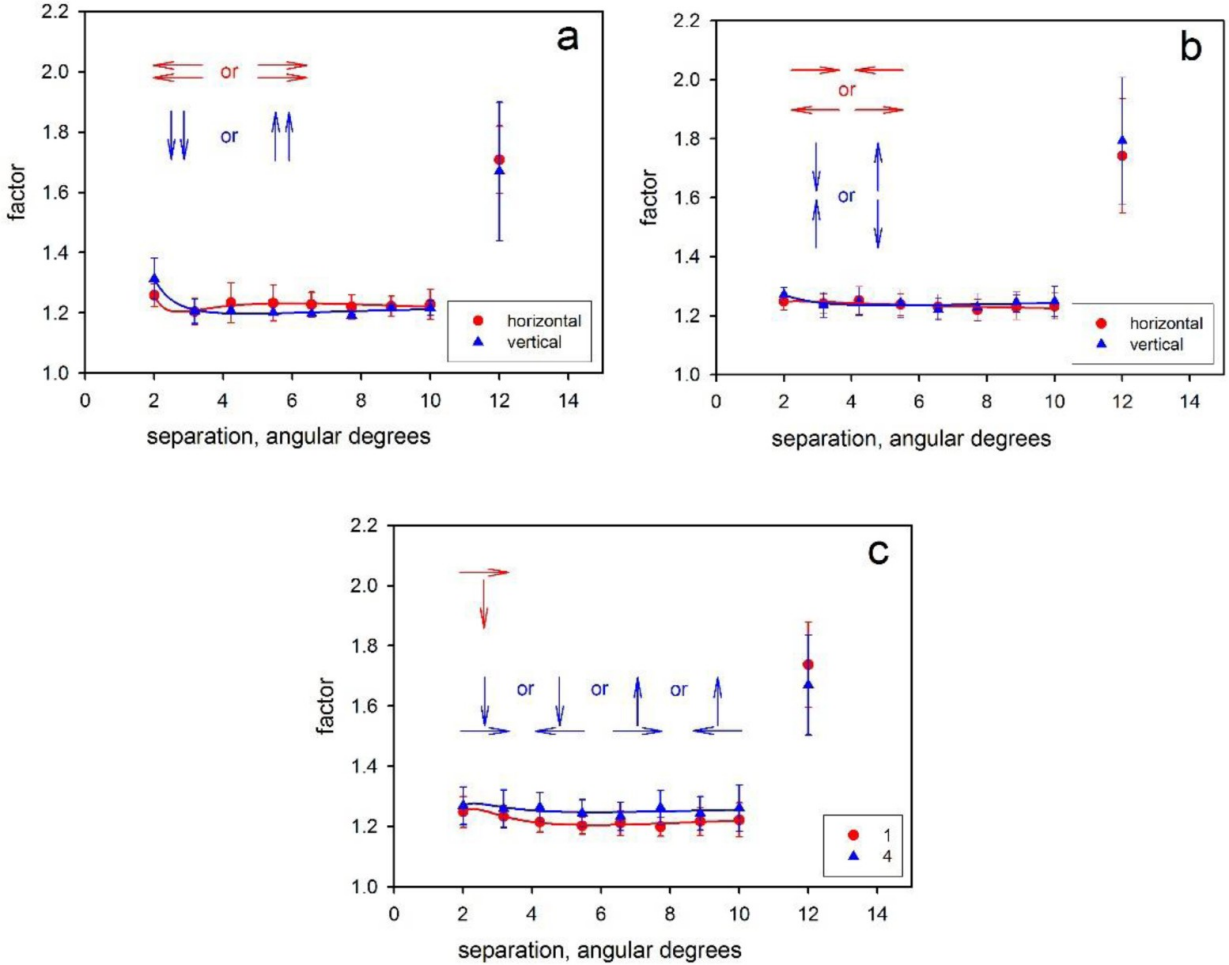
Results from six separate experiments on speed discrimination, using a roving procedure. **a**: The dots move in the same direction, either on a horizontal axis (red points) or a vertical axis (blue points). (Experiments 1 & 2). **b**: The dots move in opposite directions, either on a horizontal (red points) or a vertical axis (blue points). (Experiments 3 & 4). **c:** The dots move in orthogonal directions, either rightwards vs downwards (red points) or in any of four possible combinations (blue points). Experiments 5 & 6). The ordinate in each case represents the factor by which the test speed differs from the referent speed at threshold and the abscissa represents the spatial separation of the stimulus patches in degrees of arc (*v* Fig 1A). Data are averages from 5 highly trained observers. Error bars represent the standard errors of the mean. In each panel, the rightmost points represent an ‘absolute judgment’ condition, where the computer randomly suppressed one of the two stimulus patches.

Fig 2A shows data for experiments 1 and 2 in which the two subsets of dots moved in the same direction, either leftward or rightward (1) or upwards or downwards (2). For the horizontal axis, a repeated-measures ANOVA (excluding the data for the absolute judgment condition) shows no significant effect of the spatial separation of the discriminanda (Table 1). The data reveal no marked deterioration of discrimination as the stimuli are increasingly separated, and this remains true even when the midpoints are 10 degrees of arc apart and when, on most trials, the two patches must fall in opposite hemifields.

**Table 1:** Results of repeated-measures analyses of variance for the separate experiments 1–6.

Experiment	*df*	*F*	*p*
1	1.424	2.71	0.154
2	2.747	5.481	0.017*
3	1.993	0.722	0.515
4	3.055	1.477	0.269
5	1.765	1.056	0.387
6	1.975	0.427	0.664

In panel *a* of Fig 2 and some subsequent plots, there is a hint that thresholds are *higher* when the patches are juxtaposed than when the separation is slightly greater, a result that is reminiscent of the gap effect seen for the analogous case of chromaticity discrimination [44,45]. At a parafoveal eccentricity of 5°, the minimal spatial separation of our stimulus patches (2° center-to-center) would be within the range of ‘crowding’ according to the celebrated rule of Bouma [46]; and the elevation of thresholds could plausibly be attributed to spatial averaging or pooling of local signals [e.g. 47,48,49,50]. The effect is particularly clear for motion on the vertical axis and is likely to explain the marginal significance of spatial separation in the ANOVA for this axis (Table 1). It recalls the curious finding of Richards and Lieberman [51] that some observers are blind to differences in speed between adjacent, parafoveal arrays of dots moving in the same direction. It is interesting that the impairment for adjacent arrays is found in the present case even though an independent cue–color –differentiates the two arrays: The chromatic difference appears not to prevent compulsory pooling of motion signals.

In further experiments, we examined four cases where the two patches move in different directions:

3 The two patches move vertically but in opposite directions
4 The two patches move horizontally but in opposite directions
5 One patch moves downwards and the other moves rightwards
6 The patches move orthogonally (as in 5), but there are four possible combinations of axis and direction (horizontal left or right; vertical upward or downward).

The average results for these further experiments are shown in panels b and c of Fig 2. In all cases, thresholds exhibit a similar relationship to spatial separation: There is little change in the precision of the comparison as the distance between the discriminanda increases. Repeated-measures ANOVAs show no significant effect of separation in any of these experiments (Table 1).

The absolute levels of performance are similar whether the stimuli move in the same (Fig 2A), in the opposite (Fig 2B) or in orthogonal directions (Fig 2C, red circles). It would be improper to perform statistical tests to compare these conditions, since the data are drawn from what are formally different, non-concurrent, experiments; but we note that the observers, the procedures and the apparatus were the same for all experiments, and it is clear that thresholds vary little. The one exception to this consistency of thresholds is seen in the comparison of Experiments 5 and 6 (Fig 2C): This difference perhaps arises because four types of trial are randomly intermixed in Experiment 6 whereas in Experiment 5 there is only a single type.

In so far as discrimination thresholds are independent of spatial separation, our results are consistent with those of Nishida and colleagues [52], who found that temporal phase discrimination for motion was excellent for stimuli separated by as much as 100 deg. (However, their temporal phase task proved much more difficult when the alternating motions were orthogonal–vertical and horizontal–something not observed for our speed-discrimination task (Fig 2C)). More generally, our finding that observers can compare the speeds of well separated arrays is consistent with the ability of observers to recognize global motion in a distributed array[53], with the evidence for cooperative processes between motions in different parts of the field [54] and with the important role of bilateral optic flow in balance and vection [31,55].

In each of Experiments 1–6, the additional condition where one of the two stimuli is randomly suppressed (rightmost data points in Fig 2) yields much higher thresholds, confirming that observers are attending to both stimuli in the main conditions and not making absolute judgments of one stimulus (see Introduction for the need for this control).

**Fig 3:**
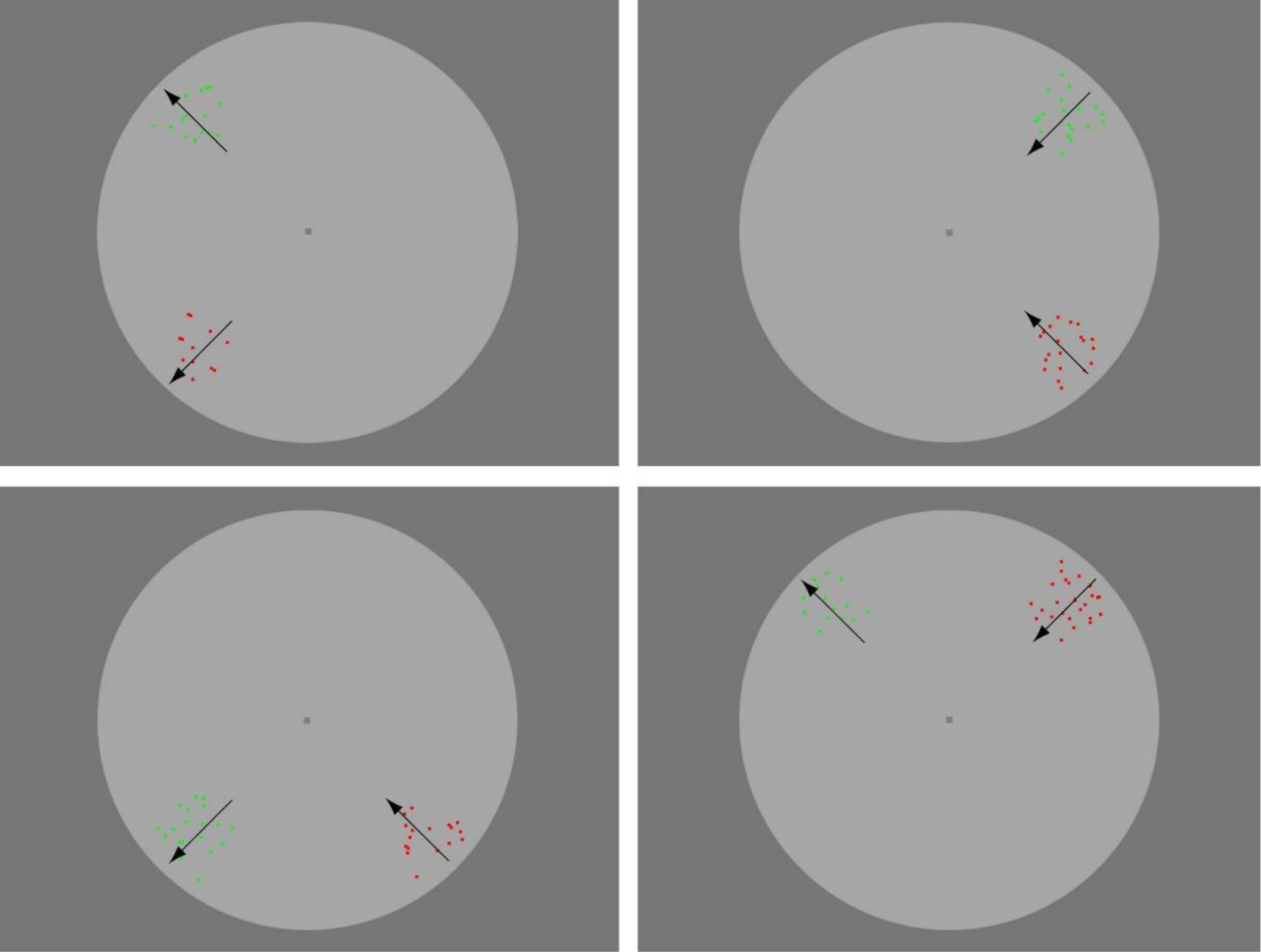
Stimulus arrangements for experiment 7. The two arrays of moving dots can fall, with equal frequency, within the left, right, upper or lower hemifields. The upper two panels represent cases where the discriminanda are both delivered to the same cerebral hemisphere and the lower panels represent cases where the two arrays are delivered to opposite hemispheres. The black arrows show examples of directions of motion. The motion is always radial and along a 45° axis that runs through the fixation point, but the direction of motion is randomly centripetal or centrifugal for any given array on any given trial. Note that each quadrant of the field is probed with equal frequency and in random sequence.

In a seventh experiment, we asked explicitly whether performance was better when the two arrays were delivered initially to the same cerebral hemisphere, compared to the case where one array falls in the left hemifield and one in the right (Fig 3). The paired arrays always fell symmetrically within one hemifield: left, right, upper or lower. The former two conditions measure within-hemisphere comparison and the latter two measure between-hemisphere comparisons. The direction of motion was always along a radial line through the fixation point, but was randomly chosen to be centrifugal or centripetal for each array on each presentation. All four hemifields were probed in the same block of trials, but separate staircases were maintained for each. A deliberate feature of the design of this experiment is that a given quadrant of the display (Fig 3) is probed equally often in comparisons that are within-hemisphere and that are between-hemisphere. So any local variation in speed sensitivity contributes equally to the two types of comparison. 19 participants completed this brief experiment, each being tested for at least 6 separate runs.

With respect to our central question–the relative advantage of intra-hemispheric vs inter-hemispheric processing–the outcome of the experiment is difficult to predict in advance. On the one hand, in the between-hemisphere condition, it can be argued that the transmission from one hemisphere to the other requires at least one additional synapse and thus the representation may be degraded and delayed [56,57]. On the other hand, there is striking evidence for a between-hemisphere advantages in reaction times when discriminanda are distributed either between or within hemifields [e.g. 58,59,60]; and in accuracy of tracking when multiple moving objects must be concurrently tracked either in one or both hemifields [e.g. 61,62]–results that are usually explained in terms of a limited processing resource that is independently available to each hemisphere.

A paired 2-tailed *t*-test showed no significant difference between the conditions where the two arrays were delivered to different cerebral hemispheres (i.e. when the discriminanda were presented in the upper or lower hemifield) and those conditions where they were delivered to the same hemisphere (i.e. when the stimuli were presented in the left or right hemifields): *t*(18) = 1.128, *p* = 0.274. The average factors by which the discriminanda differed at threshold were 1.38 (SD = 0.138) for the between-hemispheres case and 1.40 (SD = 0.15) for the within-hemisphere case–a (non-significant) difference in the wrong direction for any hypothesis that envisaged degradation of signals in transmission across the corpus callosum (see Discussion).

A perceptual advantage for the lower versus the upper visual hemifield has been reported for some motion discriminations [e.g. 63,64,65] and there is a higher density of ganglion cells in the superior parafoveal retina than in the inferior [66]. We therefore analyzed the subsets of thresholds for cases where both discriminanda fell in the upper field or both in the bottom. There was a small advantage in threshold in favor of the *upper* visual field (the average factors being 1.36 *vs* 1.40) but this difference was not significant (*t*(18) = 1.84, *p* = 0.082). However, we record that a corresponding analysis for left versus right hemifields showed a marginally significant advantage for the left: *t*(18) = 2.52, *p* = 0.021), the factors at threshold being 1.38 and 1.42.

## General discussion

We asked how well the human observer can compare the speeds of stimuli that are briefly presented at distant points in the visual field. There is little deterioration in precision even when the stimuli are as much as 10° apart; and thresholds are similar whether the two discriminanda are initially presented to the same cerebral hemisphere or are presented to opposite hemispheres.

Also relevant to our underlying question is the fact that the absolute values of the psychophysical thresholds are similar whether the discriminanda are moving in the same or in different directions (Experiments 1–5). An analogy here may be drawn with the classical findings of Burbeck and Regan [67] that orientation discrimination was equally acute whether or not the discriminanda were of the same spatial frequency, and conversely that thresholds for discriminating spatial frequency did not depend on whether or not the stimuli had the same orientation. In the case of speed, Verghese and McKee [68] found that thresholds for discriminating the speeds of arrays of dots moving in opposite oblique directions were similar to those for arrays moving parallel to one another, above and below a horizontal boundary (their Fig 2). Manning and colleagues [69] did find some effect of whether different directions were being compared; but the effects were modest and were restricted to some particular pairs of component directions. A possibly critical difference between their study and the present study is that our long-serving observers in Experiments 1–6 received feedback on every trial, and probably were performing near the limits that the human visual system can achieve, whereas Manning *et al* tested more participants for shorter time and without feedback.

In everyday life, human beings regularly have occasion to compare visual stimuli that fall at different points in the visual field; and in many such tasks, sensorimotor or cognitive feedback provides a basis for calibration over time. But perhaps it is because these perceptual comparisons are so natural and effortless that cognitive scientists have seldom asked how they are performed (or indeed, how the calibration is maintained). Our primary purpose in this paper is to define experimentally what is to be explained in the case of speed and to draw attention to the absence of any explicit neural model. However, it is of interest briefly to compare two generic classes of explanation.

### (a) Dedicated comparator neurons. The problem of imprisoned information

To account for the present results on speed discrimination (and analogous results for hue and spatial frequency), we might suppose that there does indeed exist a large population of dedicated and hardwired comparator neurons, one for each possible pair of positions in the visual field and for each of a growing list of sensory attributes–populations of cells that are still to be discovered by electrophysiologists. A critical reader might point out that receptive fields become larger at higher levels of analysis in pre-striate cortex and may extend substantially across the midline [19,24,70,71,72,73,74]; and so, it might be argued, a relatively small population of pre-striate cells might suffice and each such cell might collect the decisions of local comparators at intermediate stages of a neural net. However, if a cell is to subserve one of our comparison tasks, it is not enough that it should integrate inputs for a given sensory attribute over a large area. Rather, it must signal the difference, or the ratio, of the values of the stimulus attribute in two specific, local, and arbitrarily chosen regions of its receptive field; it must preserve the sign of the difference; and–in the present case of speed discrimination–it must be indifferent to the direction of the motion. Such prestriate cells might in principle exist, but, to our knowledge, neurons with these properties have never been described. ‘Collector units’ that integrate a particular feature over a large receptive field have been postulated in models of crowding and texture perception, but it is usually assumed that they explicitly do not preserve the exact localities from which their individual inputs originate [see ref 49].

**Fig 4:**
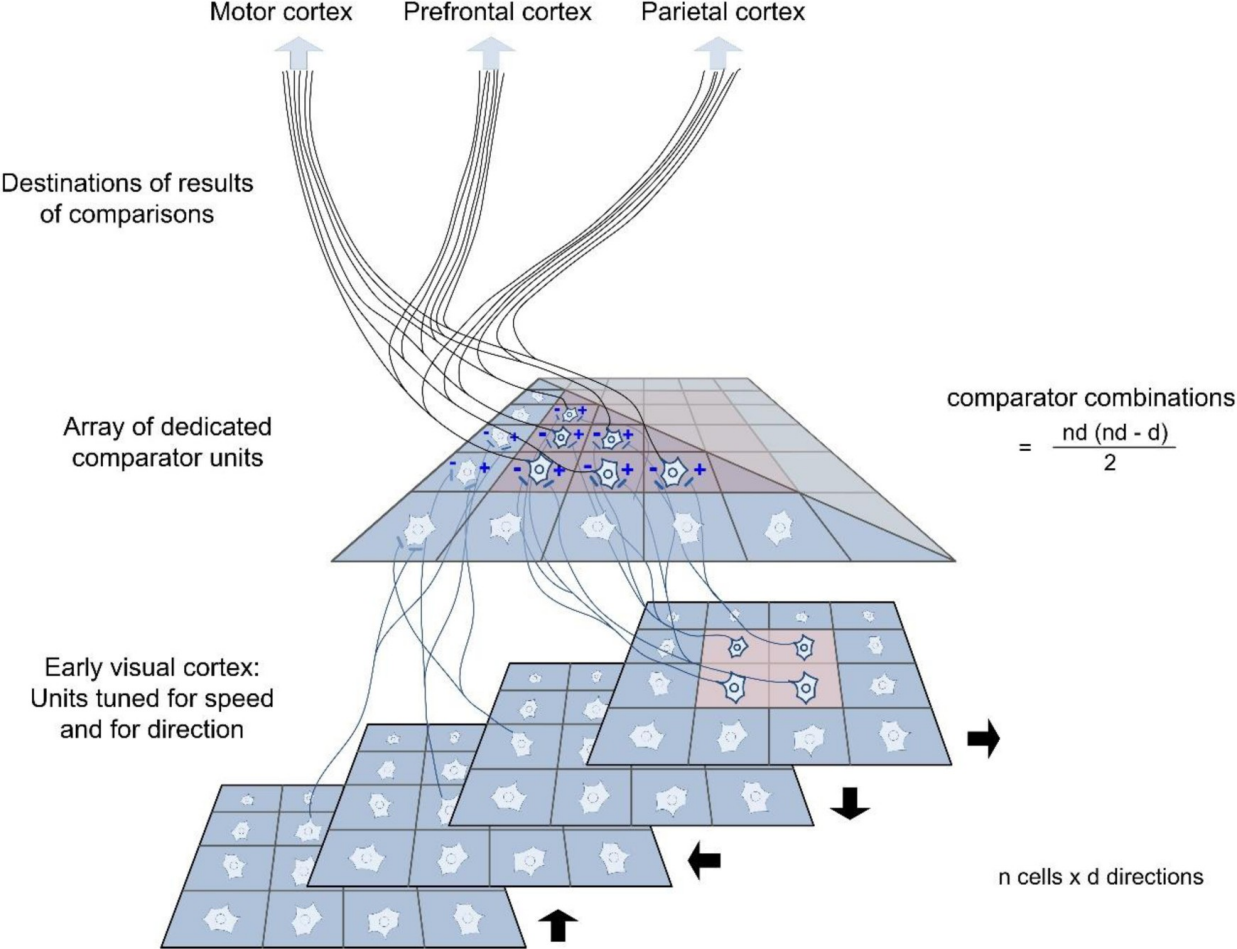
An (implausible) model in which comparisons are performed by dedicated ‘comparator units’. At the lower level, representing early visual cortex, cells that are tuned for speed and also for direction draw their inputs from local areas of the visual field (these cells might themselves derive precise estimates of speed by comparing the outputs of a small number of lower-level, broadly tuned units. See e.g. Smith and Edgar [75], Hammett et al [76], Manning et al [69]). At the upper level, there is a dedicated comparator unit for each possible pairing of cells at the first stage (the formula to the right indicates the number of comparator units that is required). A further array of dedicated projections is then needed to convey the results of any comparison forwards to any other part of the brain that might need the information.

Fig 4 sketches the combinatorial explosion of comparator cells and connections that would be required if there were a dedicated comparator cell for each pair of local positions in the field and if the array of comparators were replicated for different combinations of direction (We use 'combinatorial explosion' not in the mathematical sense but in the simple sense that is used in communication science, to indicate the increase in the number of connections required as the number of possible pairs in the network increases. See Fig 4). For the purposes of the illustration, we assume at the lower level an array of local detectors, speed-sensitive but also specific for direction and for a local region of the visual field. Multiple higher-order ‘comparator units’ draw opposed inputs from every possible pairing of lower-order units, extracting either the ratio or the difference of the speed signals.

Two problems immediately present themselves. The first is what we could call the problem of ‘junk mail’: (Unless some additional control apparatus is postulated) at any moment each unit active at the lower level is broadcasting largely unwanted action potentials to every comparator unit with which it is connected; and each action potential has a significant cost in energy [77].

The second problem is the bulk of connections and of comparator neurons that are required if comparisons of speed are performed by dedicated comparators of this kind. Setting aside the necessary white matter, it is possible to make an order-of-magnitude estimate of the bulk of neurons required. In the present experiments, we have probed sensitivity at only one, arbitrarily chosen, set of loci in the near periphery. However, motion sensitivity extends to the very margin of the visual field [e.g. 78], albeit that sampling areas become larger with eccentricity. Sackitt and Barlow [79] suggest that each hypercolumn in cortical Area 17 corresponds to an independent region of the field (varying in size according to a cortical magnification factor) and they estimate the total number of hypercolumns (*n*) in Man as ~ 5300. If we assume that *d*, the number of directions independently represented in each hypercolumn, has a value of 4, then the total number of comparator neurons needed for this task alone would be *nd*(*nd*-*d*)/2 or 2.247 x 10^8^ (see Fig 4). This is a conservative estimate: We have assumed that each comparator neuron can signal speed ratios greater or lesser than 1.0; we have also assumed that each individual comparator can handle the full range of speeds; and we have assumed that all possible directions are represented by just four lower-order detectors. The number of neurons in one cubic millimeter of human visual cortex is of the order of 4 x 10^4^ [80]. So the postulated comparator neurons for speed would occupy a cortical volume of ~5600 mm^3^. Similar volumes of comparator neurons would be required for each of several other attributes of the visual image.

There is a deeper issue. For models of this kind only postpone the problem. If any arbitrary sensory comparison relies on the signal of a dedicated comparator neuron, then the information is ‘frozen’ in the activity of that cell. The information may be required by many different modules elsewhere in the brain, but the only way that it can be delivered is by a further array of dedicated connections, thus multiplying the original problem. This is a general difficulty with all theories that suppose that entities (words, faces, concepts, as well as the outcomes of sensory comparisons) are represented centrally by the activity of single neurons–the ‘cerebral fibrils’ of Charles Bonnet [81], the ‘gnostic units’ of Konorski [82] or the ‘grandmother cells’ of Lettvin [83]: Information is imprisoned in the cell. ‘Ensemble’ or population coding–a distributed representation–does not solve this problem, since it too requires an array of dedicated connections to transmit the information onward. The information is again imprisoned–frozen in the ensemble. Sooner or later, neural apparatus is needed to extract and identify the pattern of activity embedded in the ensemble.

### (b) A cerebral bus?

In an alternative class of models of sensory comparison, local information about stimulus attributes would be transmitted to the site of comparison in an abstract form and over a shared ‘cerebral bus’, where the same physical substrate carried different information from moment to moment, as in many man-made communication networks, such as the Internet. The site of comparison might be the prefrontal cortex [84,85] and information about visual stimuli might be carried there by the inferior occipital-frontal fasciculus. Associated with each message would be codes that represented the addresses of the source and the destination. By analogy with the ‘object files’ of Anne Treisman [54 pp 123–124,^86^], the message would include the position of the stimulus as just one of its several attributes, along with speed, direction, color etc. (This would be in contrast to coding at early stages of the visual system, where all the evidence suggests that spatial coordinates are coded by labeled line, i.e. by which neuron is active.)

**Fig 5:**
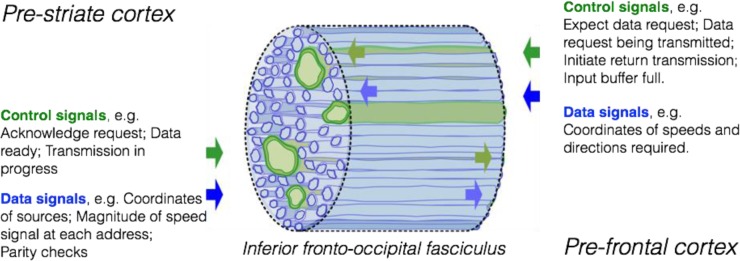
A cross-section of part of a ‘cerebral bus’. This might be, say, part of the inferior fronto-occipital fasciculus, delivering information from different parts of the visual field to the prefrontal cortex. In all white-matter tracts there is a range of axon diameters, and in this illustrative example (of what is a large generic class of models) we assign *Control* signals to the minority types of cell with large axons and we assign *Data* signals to the many smaller axons. Control signals sub-serve the ‘handshaking’ between transmitter and receiver that has proved a necessary feature of man-made communication networks [88] but is seldom discussed in the context of brain networks.

In discussions of inter-hemispheric communication, it is often suggested that there will be potential degradation at an extra synapse when the information is transmitted across the corpus callosum [56]. Yet our Experiment 7 shows that thresholds are not significantly increased when the discriminanda are initially delivered to opposite hemifields. This is not unexpected if in fact the two representations are independently transmitted to the prefrontal cortex for collation. An early DTI study suggested that the inferior fronto-occipital fasciculus of each hemisphere contains not only a component that originates in the ipsilateral occipital lobe but also a component that originates in the contralateral occipital cortex, passes through the posterior corpus callosum, and joins the ipsilateral fronto-occipital fasciculus [87; Fig 5B and 5C]. If this pathway is confirmed and if judgments of speed are made in the prefrontal cortex, then the same number of synapses may be involved in within- and between-hemisphere comparisons.

If the brain’s white-matter tracts do consist in more than dedicated connections that transmit signals between fixed points, then many interesting technical questions arise that would be asked about any man-made communication network [88] but which are curiously little discussed with respect to the brain. For example: Does data transmission occur in fixed packets or is it continuous? Are addresses encoded separately from data (e.g. in parallel fibers of different diameter)? How is the speed of transmission matched to that of the receiver and does the failure of such handshaking lead to pathologies? What are the protocols for error checking? Can packets be directed by alternative routes, as on the Internet?

Particularly salient is the question: *Is information transmitted only on demand*? We raised in the last section the energetic cost of freely broadcasting sensory information in case it is ever needed. On the Internet specific information is requested, and is returned, from the relevant address. In the brain, of course, this would correspond to selective attention–a process that would lose some of its mystery if it is an intrinsic part of the operation of a communications network.

Since axonal transmission is slow and since action potentials are limited in their maximal frequency and in their temporal precision, it is likely that a ‘cerebral bus’ will be much more parallel in its architecture than are current man-made communications networks, such as the Internet, where fiber optics allow high rates of serial transmission. So Fig 5 –where we sketch the type of activity that might occur on a cerebral bus when the present psychophysical task is performed–is very loosely inspired by the types of protocols found in parallel architectures (such as that of a ‘Small Computer System Interface’ (SCSI)). In this, strictly illustrative, model, the different types of signal (addresses of source and destination, data, transmission control protocols) are carried in parallel by axons of different diameter. In most of the white-matter fasciculi of the human brain, there is a large range of axon diameters, and thus a large range of transmission speeds [89,90]. Yet only occasionally has the explanation for this striking diversity been discussed [91]. We suggest that the control signals might reveal themselves more readily to single-unit recording than does the code in which the actual data are carried. We are not suggesting that the ‘cerebral bus’ resembles in any strong sense a SCSI bus, but man-made communications networks offer a guide as to the protocols and the control signals that are likely find their analogues in the brain [88].

In a further important way the central white-matter tracts of the brain may differ from a man-made system such as the Internet. It seems plausible, for example, that the inferior fronto-occipital fasciculus might have features that optimize the transmission of visual data, and that the arcuate fasciculus might have features favoring phonological and syntactic information–even though in each case the information is in abstract form. In this case, the data code may be specific to a particular category of information and not truly independent of the tract that is carrying it. On the other hand, the ARPANET, the military forerunner of the Internet, was explicitly designed to withstand damage to individual nodes, in that any information could be sent by any route, by means of packet switching [88]; and it is a nice question as to how far this is possible in the human brain. Some evidence that codes can be sent by more than one route comes from the recovery of interhemispheric transmission not only in cases of agenesis of the corpus callosum but in adults who have suffered severe damage to that tract.

## Conclusion

The distinction that we make above is that between a *neural net* (where information is embodied in the structure of the net, in its connections and their weights) and a *communications network* (where the data transmitted by the same physical substrate vary from moment to moment). This distinction can be seen in the much larger context of the classical debate between Connectionism and Symbolic AI [92].

It seems unquestionable that parts of the cortex do behave as a neural net–all the evidence, for example, suggests that this is the case for early stages of the visual system–but is the more central communication between cortical regions best described in the same terms? What class of model offers a more appropriate account of our psychophysical results? Given the finding that comparisons of speed are made with similar precision whatever the spatial separation of the stimuli, given that the judgments are of similar precision whether the stimuli move in the same or in opposite or in orthogonal directions, given that there is no impairment when stimuli initially arrive in different cerebral hemispheres, and given the bulk of neurons that would be required if this task, and similar sensory tasks, were performed by an array of dedicated comparator units, we favor an explanation in terms of a communications network. The distinction we make for purposes of exposition is, of course, an artificially dichotomous one: The two generic classes of model may well have intermediate forms.

Owing to the absence of physiological data, our concept of the cerebral bus is not yet at a level of detail that would allow computational modeling; but we have presented a series of psychophysical results that are compatible with the hypothesis and we have made two further suggestions of how the hypothesis could be tackled empirically: (i) By identifying electrophysiologically a subset of fascicular axons that carry stereotyped control signals; and (ii) by examining histologically the circuits present at the terminations of fasciculi. And we hope our discussion of the issue may encourage colleagues to suggest further tests.

Research such as the Human Connectome Project has recently told us much about cerebral connectivity–that is, about the *structure* of local and large-scale networks in the brain as represented by nodes and edges in graph theory [93,94]. Now is the time to raise complementary questions about the format and the protocol of information transmission over the great white matter tracts of the brain–questions that are largely unasked. Our purpose in this paper is not so much to answer such questions as to prompt interest in these fundamental issues.

## Methods

### Observers

The same five observers (3 female) took part in the preliminary experiments and the main experiments 1–6. All observers were highly trained on this and similar tasks. Observer 3 was tested in St Petersburg: Her data (see S1 Fig) are closely similar to those of the remaining observers tested in Cambridge. The data for Experiment 7 were collected wholly in Cambridge and there were 19 observers (15 female).

### Ethics statement

The experiments in both laboratories were approved by the Psychology Research Ethics Committee of Cambridge University (PRE.2008.35) and participants gave informed consent.

### Apparatus and stimuli

The experiments were performed in parallel in Cambridge and St Petersburg, using the same programs and near-identical apparatus. The display in each case was a Mitsubishi Diamond Pro 2070 22-inch CRT set at a resolution of 1024 x 768 pixels and 100 Hz and controlled by a Cambridge Research Systems (CRS; Rochester, Kent, UK) graphics board (model VSG2/5 in Cambridge, ViSaGe in St Petersburg). Monitor outputs were linearized with a silicon photodiode and the spectral power distribution for each gun at maximal output was measured with a JETI spectroradiometer model Specbos 1201 (JETI Technische Instrumente GmbH, Jena, Germany).

The typical arrangement of our stimuli is illustrated in Fig 1A. The discriminanda were two arrays of pseudo-random moving red and green dots and the observer’s task in all experiments was to report which array was moving faster. (Color is used here only to simplify the observer’s task.) The midpoints of the two target arrays fell at different positions on an imaginary circle centered at the fixation point, so that their eccentricity was constant at 5 degrees of visual angle. The width of the stimulus sectors was 2° at their individual centers. The midpoint of the two sectors took a different random position on the circle from trial to trial, and the separation of the sectors differed between experimental runs (preliminary experiments) or between experimental blocks (main experiments): At one extreme, the stimulus patches were touching and at the other extreme they were separated by a visual angle of 10° and thus lay on a diameter of the imaginary circle.

Each stimulus dot consisted of a square of 4x4 pixels, which subtended 4.38 angular minutes at the eye. The stimulus dots were anti-aliased to allow displacements of less than 1 pixel between frames. The CIE *x*,*y* chromaticities of the red and green dots were 0.5097, 0.4356 and 0.3324, 0.5729 respectively; their luminance was 38 cd^-2^; and they were presented on a steady white background (metameric to Illuminant D65) of luminance 10 cd^-2^. In order to construct the array of dots, we began with a regular array of dots separated by 25 pixels (27.4 angular minutes) and then randomly jittered the position of each dot within its cell. To provide a continuous supply of dots, the conceptual array of dots filled the full screen, but only dots geometrically within the specified sector were displayed on a given frame. Two such conceptual arrays were maintained concurrently, one moving at the reference speed on a given trial and one at the test speed. Within each array, to prevent the use of path length as a cue, dots had a limited lifetime in that we constrained the mean path length to be the same in both arrays at any given reference speed. Specifically, the mean path length was the distance in pixels travelled by the reference dots in 9 frames; and the test dots were limited to the same mean path length in pixels. In addition, for any given dot, the path length was randomly jittered around the mean so that its actual value in pixels corresponded to the distance travelled by the reference dots in 9±6 frames. Whenever a dot died anywhere in each conceptual array, a new dot was created at a mirror-image position on the screen, so as to maintain the overall density of dots.

The two arrays appeared simultaneously for 180 ms (18 frames); this duration is short enough that observers cannot move their eyes from one stimulus to another during the presentation. Viewing was binocular from 114 cm and the room was otherwise dark.

For Experiment 7, where we wished explicitly to compare hemifields, we introduced additional arrangements to maximize the symmetry of the testing situation [95]: a chin and forehead rest was used to control the observer’s viewing position, a circular aperture mask was mounted in front of the display, and the computer was placed behind the display rather than to one side. In this experiment, the two stimulus arrays always fell within one hemifield (upper, lower, left, right) and the separation was always 7 degrees of visual angle (Fig 3). Motion was always radial, towards or away from fixation, but was randomly centripetal or centrifugal, independently for each of the two arrays.

### Procedure

As a preparation for the roving procedure of our main experiments, we carried out preliminary experiments to determine a range of speeds where the Weber fraction for speed discrimination was relatively constant. In these experiments, several reference speeds were tested, without jitter, and the measurements were repeated for 3 different spatial separations. The separations were tested in different experimental runs; and within each run, there were eight different speeds, each in a separate block of trials. On a given trial, one of the two arrays, chosen randomly, was set to the referent speed and the other was set to a higher, variable speed. Thresholds were measured by a 2-alternative forced-choice method: The observer indicated by pushbuttons whether the red or the green array was moving faster, and received auditory feedback after each response. The ratio of the variable speed to the referent was adjusted by a staircase procedure that tracked 79.4% correct [96]. The step size was 10% of the difference between variable and test. Data from the first 5 reversals of the staircase were not used, and the subsequent 10 reversals were averaged to give an estimate of the threshold. For each observer, 6 independent estimates of each threshold were collected across different experimental days. The first estimate was discarded as practice in all cases and the plotted points are thus based on 5 independent measurements for each observer for each condition. For speeds in the range 3.2 to 6.8 deg/s (the range used in the main experiments), the 95% confidence limits in the preliminary experiment were equivalent to a 4% variation in threshold (S1 Fig) and so the same formal structure of data collection was adopted in all the main experiments.

For the main experiments 1–6, eight spatial separations were tested in separate blocks within one experimental run. Within each block, thresholds were measured by a single staircase, but the speed of the referent stimulus was jittered in the range 3.2 to 6.8 deg/s; and what was adjusted in the staircase was the factor by which the variable differed from the referent speed. There were 25 possible referents, spaced at intervals of 0.15 deg/s. In an additional block of trials (which could never be the first block in an experimental run but was otherwise randomly placed in the run), the experimental program randomly suppressed one of the two stimuli and the observer was asked to base his or her judgment on one of the two stimuli, as if the other had been present. This condition was to control for the possibility that observers were attending to only one of the two stimuli in the primary conditions (see Introduction). For each observer, 6 independent estimates of each threshold were collected across different experimental days. The first estimate was discarded as practice in all cases and the plotted points are thus based on 5 independent measurements for each observer.

In Experiment 7, the four hemifields (upper, lower, left and right) were tested in random order within a block of trials, and a separate staircase was maintained for each case. Staircases could terminate after 15 reversals but were allowed to run for up to 20 reversals, to minimize instances where observers were aware which staircases were still in play (and where attention might therefore become biased). In all cases the estimated threshold was based on the last 10 reversals.

## Supporting information

S1 Fig Left-hand panels: Results for individual observers in the preliminary experiment on horizontal motion. Error bars show ± standard error of the mean. Other details as for Fig 1B. Right-hand panels: Results for individual observers for speed discrimination as a function of spatial separation in Experiments (i) and (ii). Other details as for Fig 2A.
(DOCX)
