# Supplementary material for: How does the human visual system compare the speeds of spatially separated objects?
Source: PLoS One. 2020 Apr 30;15(4):e0231959. doi: 10.1371/journal.pone.0231959 (PMC7192430; doi:10.1371/journal.pone.0231959)
Supplement: S1 Fig — Left-hand panels: Results for individual observers in the preliminary experiment on horizontal motion. Error bars show ± standard error of the mean. Other details as for Fig 1B. Right-hand panels: Results for individual observers for speed discrimination as a function of spatial separation in Experiments (i) and (ii). Other details as for Fig 2A. (DOCX) [file pone.0231959.s001.docx]

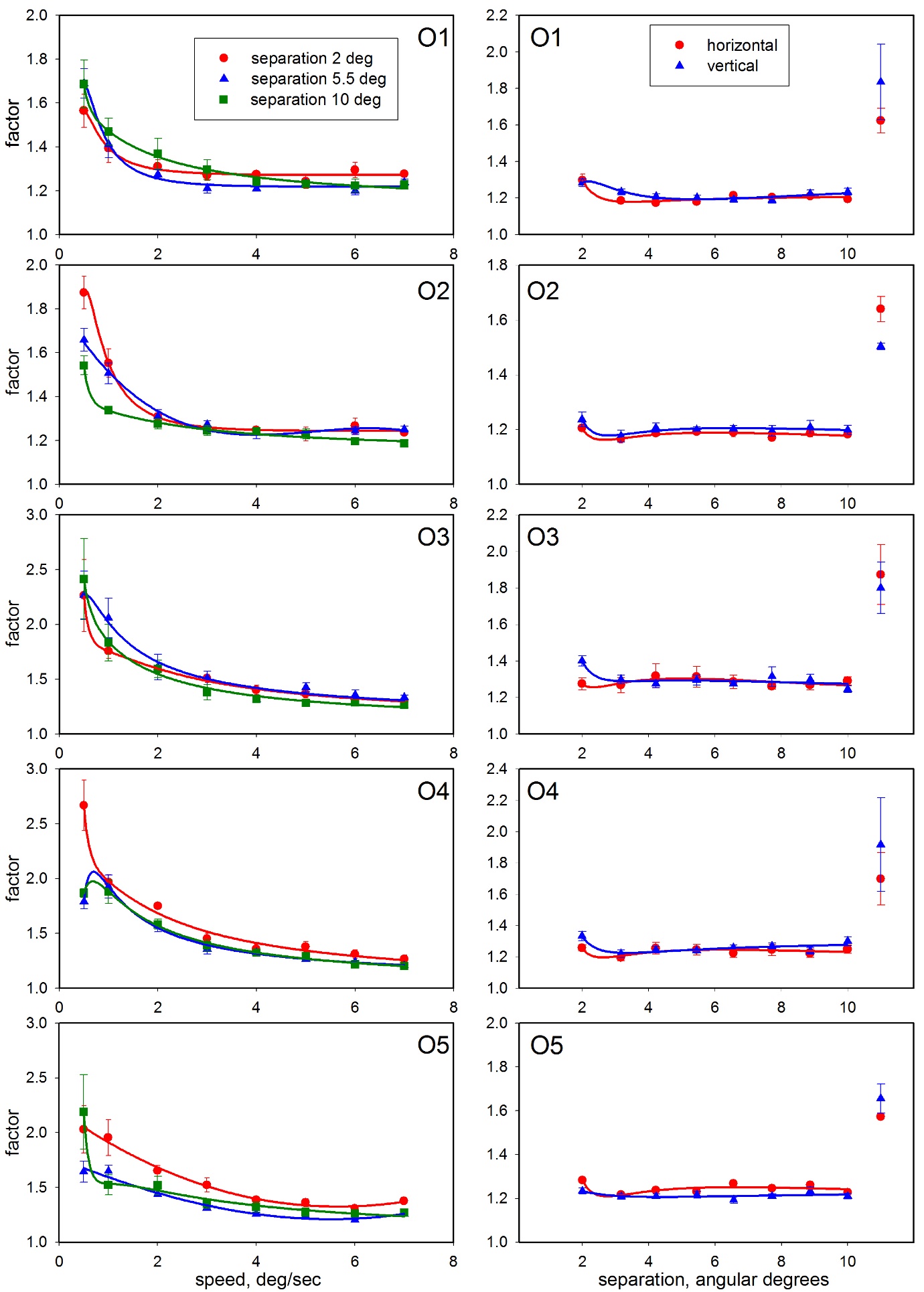


Supplementary Figure 1. Left-hand panels: Results for individual observers in the preliminary experiment on horizontal motion. Error bars show ± standard error of the mean. Other details as for Fig 1b. Right-hand panels: Results for individual observers for speed discrimination as a function of spatial separation in Experiments (i) and (ii). Other details as for Fig 2a.
